# Plasma Exosomes as Markers of Therapeutic Response in Patients with Acute Myeloid Leukemia

**DOI:** 10.3389/fimmu.2014.00160

**Published:** 2014-04-10

**Authors:** Chang-Sook Hong, Laurent Muller, Theresa L. Whiteside, Michael Boyiadzis

**Affiliations:** ^1^Department of Pathology, University of Pittsburgh Cancer Institute, University of Pittsburgh School of Medicine, Pittsburgh, PA, USA; ^2^Department of Immunology, University of Pittsburgh Cancer Institute, University of Pittsburgh School of Medicine, Pittsburgh, PA, USA; ^3^Department of Medicine, University of Pittsburgh Cancer Institute, University of Pittsburgh School of Medicine, Pittsburgh, PA, USA

**Keywords:** acute myelogenous leukemia, exosomes, TGF-β1, protein levels, chemotherapy

## Abstract

**Purpose:** Exosomes isolated from the plasma of newly diagnosed acute myeloid leukemia (AML) patients have elevated protein and transforming growth factor-beta 1 (TGF-β1) contents and inhibit natural killer (NK) cell cytotoxicity (*Haematologica* 96, p. 1302, 2011). A potential role of exosomes in predicting responses to chemotherapy (CT) was evaluated in AML patients undergoing treatment.

**Experimental Design:** Plasma was obtained from AML patients at diagnosis (*n* = 16); post-induction CT (*n* = 9); during consolidation CT (*n* = 10); in long-term remission (Lt-CR, *n* = 5); and from healthy volunteers (*n* = 7). Exosomes were isolated by size-exclusion chromatography and ultracentrifugation. The exosomal protein, soluble TGFβ-1 levels (ELISA), and the TGF-β1 profiles (western blots) were compared among patients’ cohorts. The results were correlated with the patients’ cytogenetic profile, percentage of leukemic blast, and outcome.

**Results:** At diagnosis, protein and TGF-β1 levels were higher (*p* < 0.009 and *p* < 0.004) in AML than control exosomes. These values decreased after induction CT (*p* < 0.05 and *p* < 0.004), increased during consolidation CT (*p* < 0.02 and *p* < 0.005), and normalized in Lt-CR. While TGF-β1 and protein levels tracked one another, TGF-β1 pro-peptide, latency-associated peptide (LAP), or mature TGF-β1 differentially decorated exosomes isolated before, during, and after CT. Only TGF-β1 pro-peptide was seen in exosomes of controls or Lt-CR patients. During consolidation CT, exosomes carried TGF-β1 pro-peptide, LAP, and low levels of mature TGF-β1. NK cell co-incubation with AML exosomes carrying all three TGF-β1 forms induced down-regulation of NKG2D expression.

**Conclusion:** Changes in exosomal protein and/or TGF-β1 content may reflect responses to CT. The exosomal profile may suggest the presence of residual disease in patients considered to have achieved complete remission.

## Introduction

Sixty to eighty percent of adult patients with newly diagnosed acute myeloid leukemia (AML) attain a complete remission (CR) with intensive induction chemotherapy [CT; Ref. (1, 2)]. However, without additional cytotoxic therapy, virtually all of these patients relapse. Post-remission therapy aims to destroy leukemia cells that survive induction CT but are undetectable by conventional methods. Ascertaining whether a patient in CR is destined to remain clinically disease free is limited by the inherent insensitivity of currently available tests for detecting residual leukemia and by the likelihood that the small area of bone marrow examined does not reflect the potential involvement of the entire, much larger, bone marrow compartment.

Exosomes are virus-size (30–100 nm in diameter) membrane-bound microvesicles that are formed within the endocytic compartments and via fusion of multivesicles bodies with the cell membrane are released into the extracellular space (3). While exosome secretion occurs under physiologic conditions, and all cells are capable of their release, tumor cells are avid exosome producers. The exosome fractions obtained from plasma of cancer patients are enriched in various immunosuppressive molecules and in proteins/glycoproteins expressed on cell membranes and/or in the cytosol of the parent tumor cells. In AML, we reported highly elevated exosome plasma levels in newly diagnosed untreated AML patients compared to the levels measured in normal controls [NC; Ref. (4)].

Based on the potential role of tumor-derived exosomes (TEXs) as mediators of tumorigenesis (5–7), we reasoned that exosome plasma levels and especially the molecular content of isolated exosomes, which are thought to mimic that of leukemic blasts, could be informative about the presence in the bone marrow of leukemic blasts that might avoid detection by conventional hematopathological assays. Therefore, studies of blast-derived exosomes in the plasma of patients at the time of initial AML diagnosis and, especially, of changes in the exosomal profile during and after consolidation CT, might substantially increase our ability to identify AML patients at the highest risk for relapse. To test this hypothesis, we isolated and studied exosomal fractions obtained from plasma of various cohorts of AML patients in a cross-sectional study. We report here that changes in total exosomal protein levels and the presence of different forms of transforming growth factor-beta 1 (TGF-β1) carried by AML exosomes reflect effects of therapy and might serve as indicators of leukemic relapse in AML patients. In addition, AML exosomes carrying an active form of TGF-β1 induced down-regulation of NKG2D expression in normal natural killer (NK) cells.

## Materials and Methods

### Patients with acute myeloid leukemia and healthy volunteers

Samples of venous blood (20–50 mL) were obtained from 16 patients newly diagnosed with AML and prior to any treatment. In addition, samples were obtained from 9 patients on day 14 after starting induction CT with anthracycline and cytarabine, 10 patients undergoing consolidation CT with a high dose of cytarabine, and 5 patients in a long-term remission following consolidation CT. Venous blood was also collected from age-matched healthy volunteers (*n* = 7). Peripheral blood was collected into heparinized vacutainer tubes, and the samples were hand-carried to the laboratory, processed, and either immediately used for experiments or aliquoted and frozen at −80°C. All subjects participating in this cross-sectional study signed an informed consent approved by the Institutional Review Board of the University of Pittsburgh. The AML patients were grouped into three cytogenetic risk categories based on published criteria (8, 9). The favorable risk category included patients with abnormalities (abn) of inv (16)/*t*(16;16)/del(16q) or *t*(8;21) without either a del(9q) or being a part of the more complex karyotype. The intermediate-risk category included patients characterized by +8, −Y, +6, del (12p), or a normal karyotype. The unfavorable risk category was defined by the presence of one or more of −5/del(5q), −7/del(7q), inv(3q), abn 11q, 20q, or 21q, del(9q), *t*(6;9), *t*(9;22), abn 17p, or the more complex karyotype defined as three or more abnormalities.

### Isolation of exosomes

Exosomes were isolated from plasma of NC or AML patients using differential centrifugation, size-exclusion chromatography on Sephadex G50 columns, and ultracentrifugation, as previously described (10). Briefly, aliquots of plasma (up to 9 mL) were centrifuged at 1000 × *g* for 10 min, filtered with 0.22 μm syringe filter unit, centrifuged again at 10,000 × *g* for 30 min, applied to an A50m column (Bio-Rad Laboratories, Hercules, CA, USA), packed with Sepharose 2B (Sigma-Aldrich, St. Louis, MO, USA), and were eluted with phosphate buffered saline (PBS). The protein content was monitored by measuring absorbance at 280 nm. Fractions between 10 and 28 mL (the void volume peak) contained >50,000 kDa proteins and exosomes. Three 9 mL fractions were collected, and after discarding the first fraction, the second and third fractions were combined, placed in a Beckman Optiseal Centrifuge Tube and centrifuged in a Beckman Optima LE-80K Ultracentrifuge (Beckman Coulter) at 100,000 × *g* for 2 h at 4°C. The pelleted exosomes were resuspended in PBS (200 μL) and analyzed using a Bio-Rad protein assay kit (Bio-Rad Laboratories, Hercules, CA, USA). The protein content of all final exosomal fractions was normalized to 1 mL plasma, and the data are presented in microgram exosomal proteins/milliliter plasma.

### Characterization of AML plasma-derived exosomes

**Figure 1:**
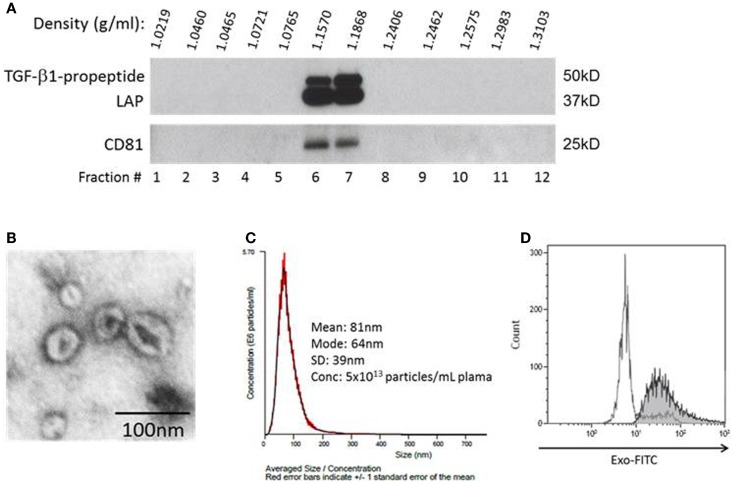
**Characteristics of exosomes isolated from AML plasma**. **(A)** A representative western blot of exosomes floated on a continuous sucrose gradient. Individual 1 mL fractions were collected and after ultracentrifugation were loaded on gels for electrophoresis. Exosomes expressing CD81 and TGF-β1 are located in fractions 6 and 7. **(B)** Transmission electron microscopy of exosomes collected from the sucrose gradient shown in **(A)** as fraction #6, negative stain with uranyl acetate. **(C)** Exosomes in fraction #6 were examined in a NanoSight instrument to determine their size and particle concentration. **(D)** Flow cytometry of AML plasma-derived exosomes (30 μg protein) captured on streptavidin and biotinylated anti-CD81 Ab-coated magnetic beads and visualized with the EXO-FITC dye. Gate was set on single beads. The white peak shows beads without exosomes, while the shaded peak shows beads carrying exosomes visualized with the EXO-FITC dye. A representative experiment of three performed with exosomes captured from different AML patients’ plasma is shown.

To show that fractions recovered from plasma as described above contained exosomes, we subjected the pellet content to continuous sucrose gradient (0.25–2.5 M) centrifugation as previously described (11) Serial 1 mL fractions at an increasing sucrose density were collected and evaluated by western blots for expression of exosomal markers, CD81 (Figure 1A) and LAMP-1 (not shown), by using antibodies specific for these proteins. In addition, isolated exosomes were visualized by negative staining with 1% uranyl acetate in water using a transmission electron microscope (Figure 1B), and their size and density were determined using a NanoSight instrument (Figure 1C). Finally, isolated exosomes were captured on 9.1 μM beads coated with streptavidin and biotinylated anti-CD81 antibody and were visualized and quantified by flow cytometry using the EXO-FITC dye (all from SBI, Mountain View, CA, USA; Figure 1D). In all procedures utilizing antibodies, isotype control antibodies were included.

### Western blots

Isolated exosomes were characterized for expression of TGFβ-1 pro-peptide, latency-associated protein (LAP), mature TGFβ-1, CD9, CD81, CD34, CD117, and GAPDH using western blots as previously described (4). Aliquots (10 μg) of isolated exosomes were lysed with Laemmli sample buffer (Bio-Rad Laboratories, Hercules, CA, USA), separated on 7–15% SDS/PAGE gels, and transferred onto PVDF membrane (Millipore, Billerica, MA, USA) for western blot analysis. Membranes were incubated with antibodies of TGF-β1, which detect TGF-β1 pro-peptide, LAP, and mature TGF-β1 (1:1000, Cell Signaling #3711; 1: 2000, R&D Systems AF-264-NA or AF-101-NA), CD9 (1:500, Abcam, ab65230), CD34 (1:2000, Abcam, ab81289) and c-kit (1:100, Abcam, ab5506), CD81 (1:200, Thermo Fisher, PA5-13582), or GAPDH (1:500, Santa Cruz, FL-335) for overnight in 4°C and then with the HRP-conjugated secondary antibody (1:5000, Pierce Chemical) for 1 h at room temperature (RT) and developed with ECL western blotting detection reagents (GE Healthcare Biosciences, Pittsburgh, PA, USA). The intensities of the bands on exposed films were semi-quantified using Image J software (NIH).

### ELISA for TGF-β1

Levels of TGFβ-1 in patients’ plasma or in exosomal fractions were quantified using a Quantikine ELISA kit purchased from R&D Systems, Minneapolis, MA, USA. Prior to ELISA, exosomes were sonicated to release membrane-bound TGF-β1 and then acidified to activate latent TGF-β1. Isolated exosome fractions were first sonicated using five 2-s bursts at 35 W in a sonicator bath (Lab-line Instruments, Melrose Park, IL, USA). Samples were then acidified with 1 N HCl (40 μL sample plus 20 μL HCl) for 10 min at RT and neutralized with an equal volume (20 μL) of 1.2 N NaOH/0.5 M HEPES. Acidified samples were diluted 1:10 and 1:20 in PBS prior to ELISA. Sonication was compared to freeze/thaw or to 10 M urea and was found to be the most efficient method for release of TGF-β1 from exosomes (data not shown). The sensitivity of ELISA was 1.7 pg/mL, and recombinant TGF-β1 purchased from R&D Systems was used as a positive control.

### Functional studies with isolated exosomes

Assays in which normal human NK cells were co-incubated with exosomes to determine down-regulation of NKG2D on the surface of these cells were previously described (4). Briefly, NK cells (1 × 10^6^) were co-incubated for 48 h with exosomes (50 μg) isolated from AML plasma. Then, flow cytometry for NKG2D expression was performed and data expressed as mean fluorescence intensity (MFI). NK cells incubated in medium were used as controls. Isolation of human NK cells from normal donor buffy coats using AutoMACS was also previously described (4). Expression patterns of TGF-β1 on the isolated exosomes, as seen in western blots, were correlated with the ability of these exosomes to inhibit NKG2D expression on human NK cells upon co-incubation. Antibodies to TGF-β (1 μg/mL, MAB240, R&D Systems) or isotype control antibodies added prior to co-incubation with exosomes were used to prevent down-regulation of NKG2D expression on NK cells. Recombinant human (rh) TGF-β1 (10 ng/mL) purchased from R&D Systems was used as a positive control.

### Statistical analysis

Data were summarized by descriptive statistics: means and standard errors (SE) for continued variables or the frequency and percentages for categorical variables. Statistical analyses were performed using paired and unpaired two-tailed Student’s *t*-test. When the data were not normally distributed (i.e., exosomal protein and TGF-β1 levels at AML diagnosis; Shapiro–Wilk normality test, *p* < 0.005), Wilcoxon–Mann–Whitney rank sum test was performed. A *p* value of <0.05 was considered to be statistically significant.

## Results

### Exosomes isolated from the plasma of AML patients

Acute myeloid leukemia exosomes isolated patients’ plasma accumulated at the density of 1.16–1.18 g/mL on continuous sucrose density gradients (Figure 1A). They had a typical “donut-like” appearance in a transmission electron microscope (Figure 1B), were uniform in size (30–150 nm in diameter) by NanoSight measurements (Figure 1C), and carried typical exosomal markers, e.g., CD81, as confirmed by western blots and also by flow cytometry of exosomes captured on beads (Figure 1D). These criteria are consistent with the characteristics ascribed to exosomes (11). Further, the exosomal fraction banding at the sucrose density of 1.12–1.18 contained TGF-β1 pro-peptide and LAP (Figure 1A). The recovery of exosomes was 5 × 10^13^ particles/mL plasma (NanoSight).

### Exosomal protein levels at the time of AML diagnosis

**Figure 2:**
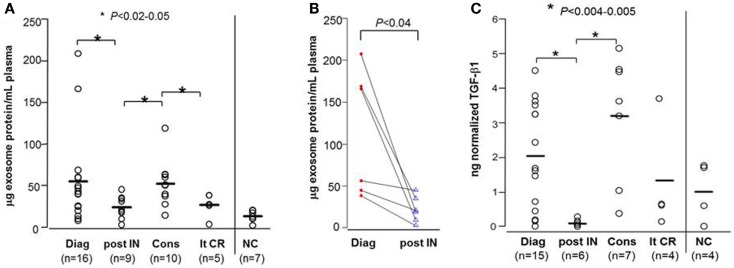
**Protein levels and soluble TGF-β1 levels in exosomes isolated from AML patients’ plasma**. **(A)** Exosomal protein levels (in microgram per milliliter plasma) for all patients in different cohorts: Diag, at AML diagnosis; post IN, 14 days after starting induction CT; Cons, during consolidation CT; lt-CR, long-term complete remission; NC, normal control. **(B)** Exosomal protein levels in serial plasma samples obtained from the same patients at AML diagnosis and 14 days after starting induction CT. **(C)** Exosomal TGF-β1 levels measured by ELISA after exosome sonication/acidification for patients in different cohorts. In this and all subsequent figures, TGF-β1 values were normalized per microgram exosomal protein in 1 mL plasma. The bars indicate mean levels.

The patients’ demographics and hematopathological characteristics at the time of diagnosis and at different time points during their leukemia treatment are presented in Table 1. The exosome fractions isolated from AML patients’ plasma at diagnosis had a considerably greater mean protein content (55.2 ± 13.8 μg protein/mL plasma) than did exosome fractions isolated from the plasma of NC (13.1 ± 2.4 μg protein/mL plasma) with *p* < 0.009 (Figure 2A). Of interest, two patients had exosomal protein levels that were highly elevated relative to the mean protein level for this patient cohort (Figure 2A). These data point to large differences in the exosomal protein content, ranging from normal to elevated, among AML patients studied at diagnosis. However, neither the percentage of blasts nor the patients’ cytogenetic profiles correlated with the exosomal protein levels at AML diagnosis. In addition, the exosomal protein levels of AML patients whose blasts were found to be reduced to <5% following a course of induction CT were not significantly different from those in AML patients with residual leukemia after a course of induction CT (78 ± 33 vs. 51 ± 24 μg protein/mL plasma at *p* = 0.5). Thus, exosomal protein levels at AML diagnosis did not appear to predict response to induction therapy.

**Table 1:** **Characteristics of AML patients included in this study**.

**AT AML DIAGNOSIS PRIOR TO TREATMENT (*n* = 16)**	
Median age (years)	64 (Range, 36–75)
**Cytogenetic risk category at diagnosis**
Unfavorable	9 (56%)
Intermediate	7 (44%)
Favorable	0
% Blasts in bone marrow at AML diagnosis	64 (Range, 38–93)
**NEWLY DIAGNOSED AML PATIENT WHO RECEIVED INDUCTION CHEMOTHERAPY (*n* = 9)**	
Median age (years)	57 (Range, 36–70)
**Cytogenetic risk category at diagnosis**
Unfavorable	7 (64%)
Intermediate	4 (36%)
Favorable	0
% Blasts in bone marrow at AML diagnosis	72 (Range, 52–93)
**AML PATIENTS IN CR DURING CONSOLIDATION THERAPY (*n* = 10)**	
Median age (years)	51 (Range, 28–65)
**Cytogenetic risk category at diagnosis**
Unfavorable	2 (20%)
Intermediate	8 (80%)
Favorable	0
% Blasts in bone marrow at AML diagnosis	53 (Range, 21–83)
**AML PATIENT IN CR IN LONG REMISSION AFTER CONSOLIDATION THERAPY (*n* = 5)**	
Median age (years)	46 (Range, 33–59)
**Cytogenetic risk category at diagnosis**
Unfavorable	0
Intermediate	4 (80%)
Favorable	1 (20%)
% Blasts in bone marrow at AML diagnosis	67 (Range, 50–82)

### Effects of CT on exosomal protein levels

Of 9/16 newly diagnosed AML patients received induction CT. Following a course of induction CT, there was a significant reduction in exosomal protein levels (to 23.8 ± 4.4 μg protein/mL plasma at *p* < 0.05; Figure 2A), concomitant with the reduction of AML blasts in the bone marrow. In six patients, serial pre- and post-induction plasma was available, and the paired data (Figure 2B) confirmed a decrease in exosomal protein levels. The exosomal protein levels on day 14 after the initiation of induction CT in patients who had a reduction of leukemic blasts to <5% was lower, but not significantly so, compared to the patients still having residual leukemia (18.5 ± 5.8 vs. 30.8 ± 7.5 μg protein/mL plasma; *p* = 0.2).

During consolidation therapy with high dose cytarabine, the exosomal protein levels in 10 patients who achieved CR was higher (52.4 ± 8.9 μg protein/mL plasma) relative to levels measured immediately post-induction CT (*p* < 0.02), reaching a mean level close to that seen at AML diagnosis (Figure 2A). Two of these 10 patients went on to receive allogeneic hematopoietic cell transplantation (allo-HCT) after two courses of consolidation CT. The remaining eight patients completed four courses of consolidation CT. Five/eight patients have subsequently relapsed. The exosomal protein levels during consolidation CT were not significantly different between the five patients who relapsed and those three who remained in CR (mean values: 58.4 vs. 47.0 μg protein/mL plasma).

Exosomal protein levels of AML patients in long-term remission (>2 years after completing consolidation CT) were not significantly different from those seen in exosomes of NC (Figure 2A). To date, with the median follow up of 48 months, none of these five patients has relapsed.

In aggregate, these data suggest that significant changes in the exosomal protein levels that occur after induction and during consolidation CT could be useful for evaluating patients’ responses to CT.

### TGF-β1 levels in AML exosomes

We have previously shown that AML plasma-derived exosomes carry TGF-β1 (4). In this study, we investigated changes in exosome-associated TGF-β1 levels in relation to therapy given to AML patients (Figure 2C). In general, TGF-β1 levels (measured after exosome sonication and acidification) tracked the exosomal protein levels but were substantially better at discriminating among AML patients at diagnosis and, especially, among patients undergoing consolidation CT (compare Figures 2A,C). As expected, TGF-β1 levels at AML diagnosis were higher than those in exosomes of NC (Figure 2C). Following induction CT, TGF-β1 levels were drastically reduced (*p* < 0.005). Importantly, exosomal TGF-β1 levels were found to be elevated relative to post-induction levels (*p* < 0.004) in patients receiving consolidation CT (Figure 2C). Levels of active TGF-β1 were especially high in four of the patients undergoing consolidation therapy (two were high risk and underwent allo-HCT, one relapsed, and one remains in remission). TGF-β1 levels in exosomes of AML patients in long-term CR were not significantly different from those of NC.

### TGF-β1 profiles in AML exosomes

**Figure 3:**
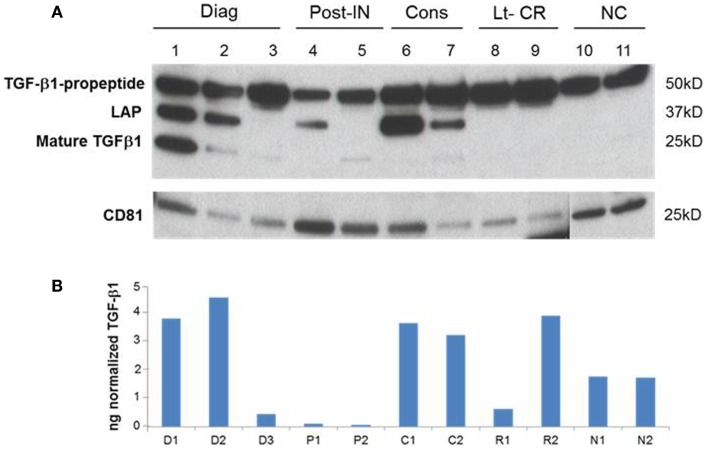
**A representative western blot of TGF-β1 profiles in AML exosomes**. **(A)** Exosomes carry TGFβ1 pro-peptide consisting of latency-associated peptide (LAP) covalently bound to the mature TGFβ1 (50 kDa), cleaved LAP (37 kDa) and mature TGFβ1 (25 kDa). Ten micrograms of exosomal proteins were separated on SDS/PAGE gel and analyzed by western blots using anti-TGFβ1 and anti-CD81 antibodies. **(B)** Active TGFβ1 levels in the corresponding exosomes following their sonication/acidification. Results are from ELISA performed as described in the Section “Materials and Methods.”

**Figure 4:**
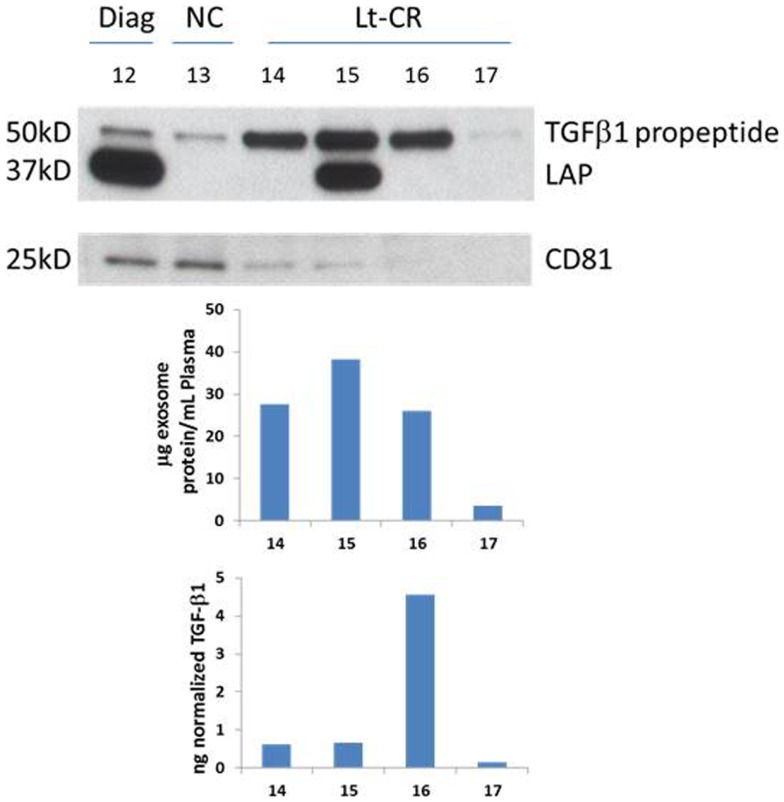
**TGF-β1 profiles of four patients in long-term clinical remission**. Western blots for TGF-β1 and CD81, an exosomal marker, were performed as described in the Section “Materials and Methods.” Few exosomes were recovered from the plasma of patient #17, as indicated by a nearly absent CD81 signal. Western blots of exosomes isolated from plasma specimens of a representative AML patient and a NC are included for comparisons. Protein levels and soluble TGF-β1 levels were measured in the same exosomes and the respective values are shown below.

A representative western blot (Figure 3A) shows that AML exosomes carry the TGF-β1 pro-peptide consisting of the LAP covalently bound to mature TGF-β1 (~50 kDa), cleaved LAP (~37 kDa) and mature TGF-β1 (~25 kDa), which under reducing conditions may dissociate into 2 × 12.5 kDa chains (12, 13). In comparison to exosomes obtained from the plasma of NC, those isolated from AML patients’ plasma carry different forms of TGF-β1 (Figure 3A). Further, exosomes isolated from patients’ plasma before, during, or after CT carry the three TGF-β1 forms (the pro-peptide, LAP or active, mature form) in distinctly different proportions. Also, these exosomes were found to contain different levels of active TGF-β1, as measured by ELISA after exosome sonication/acidification (Figure 3B). The levels of exosomal LAP expression were generally, but not always, high at AML diagnosis and also in exosomes of patients undergoing consolidation CT (Figure 3). This observation implies that exosomal TGF-β1 levels and TGF-β1 activation in exosomes might reflect the presence of residual disease. Exosomes obtained from the plasma of four AML patients in long-term clinical remission differed from each other and from NC in the quality and quantity of the TGF-β1 cargo (Figure 4).

### Functional relevance of TGF-β1 carried by exosomes

**Figure 5:**
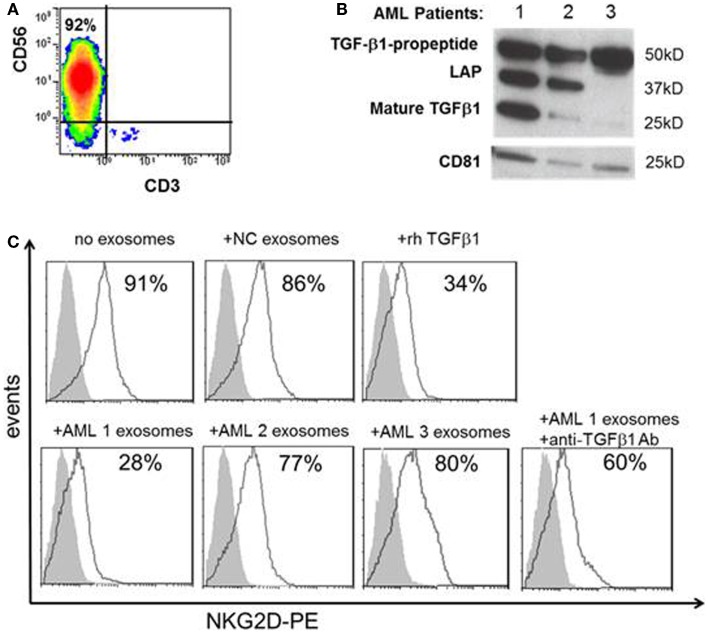
**Effects of TGF-β1^+^ exosomes isolated from AML plasma on NK cells**. **(A)** Human CD3^neg^CD56^+^ NK cells were isolated from the peripheral blood of a normal volunteer and evaluated by flow cytometry. **(B)** Western blots of TGF-β1 exosomes isolated from three AML patients, each with a distinct TGF-β1 profile. CD81 served as an exosomal marker. **(C)** After co-incubation of NK cells with exosomes isolated from AML plasma as described in the Section “Materials and Methods.” NKG2D expression (in MFI) was found to be down-regulated only with exosomes of patient #1 and rhTGF-β1. Exosomes isolated from a NC had no effect on NKG2D expression on NK cells. Anti-TGF-β1 Ab protected NK cells from NKG2D down-regulation by exosomes of patient #1. Shown is a representative experiment of three performed with NK cells obtained from different donors. The shaded peaks represent isotype controls.

To determine whether the distinct TGF-β1 profiles seen in exosomes obtained from AML patients at diagnosis and during therapy are related to its biologic activity, purified normal human NK cells (Figure 5A) were co-incubated with AML exosomes isolated from the plasma of three different newly diagnosed AML patients selected based on different exosomal TGF-β1 expression patterns in western blots (Figure 5B). Linking the different TGF-β1 western blot patterns with biological functions mediated *in vitro* by these exosomes, we observed that only exosomes of AML patient #1 carrying high levels of mature TGF-β1 inhibited expression of NKG2D in NK cells (Figure 5C). This inhibition was comparable to that mediated by rhTGF-β1, and it was reversed by neutralizing antibody to TGF-β1. These data confirm the relevance of TGF-β1 profiles in exosomes to their inhibitory activity vis a vis human NK cells, which are known to be highly sensitive to TGF-β1 (4, 14–16).

## Discussion

Despite considerable progress and a relatively high morphologic remission rate (approaching 70–80% in younger adults) with intensive CT, only 30–40% of AML patients survive 5 years after diagnosis. Many patients experience relapse, which is likely related to the presence of minimal residual disease. It is important to be able to identify AML patients who are at high risk of relapse and need intensive post-remission treatment, and patients who can potentially be cured with the currently available post-remission regimens and are likely not to need additional therapy, thereby reducing the treatment-related morbidity and mortality associated with post-remission strategies.

In patients with melanoma and other solid tumors, total protein levels of exosome fractions isolated from plasma were reported to reflect disease stage, tumor burden, response to therapy, and even survival, with patients characterized by a high exosomal protein levels experiencing more advanced disease and shorter survival (5–7). These reports provided a rationale for this cross-sectional study of AML patients at the time of initial diagnosis prior to any therapy and at different stages of their therapy, including patients in complete clinical remission. Plasma-derived exosomes are a mix of nanovesicles originating from various cells, including leukemic blasts. As exosomes are known to be abundantly produced by stressed cells (17), it seemed conceivable that in AML, exosomal protein levels could reflect the extent of disease and correlate with its relapse after therapy. Indeed, large differences were noted in exosomal protein levels between patients at AML diagnosis, but as previously reported by us (4), correlations with hematopathological, genetic, and clinical data were not significant. Nevertheless, the large differences seen in exosomal protein levels among AML patients at diagnosis are intriguing and provide a rationale for further exploration of a potential predictive value of such data. In terms of therapy-related changes in exosomal protein levels, it appears that significantly decreased protein levels in exosomal fractions after induction CT were concomitant with the reduction of AML blasts in the bone marrow. Further, in some patients receiving consolidation therapy who subsequently relapsed, exosomal proteins were elevated, suggesting residual disease at the time when leukemic blasts were undetectable in the bone marrow by conventional methods. Importantly, exosomal protein levels were not significantly elevated in the plasma of patients who achieve long-term CR, suggesting that low exosomal protein levels might be predictive of long-term disease free survival. These preliminary data suggesting that exosomal proteins have a potential predictive value in AML are obviously limited by the cross-sectional design of our study and small numbers of patients in each cohort. Only a few patients had serial samples available for the examination of changes in exosomal protein levels during CT. Yet, these preliminary data suggested that serial monitoring of exosomes is likely to be important in search for new tests with higher predictive values for disease progression/relapse or responses to CT in AML.

We and others have shown that exosomes are involved in regulating functions of immune cells (18, 19) and thereby could contribute to cancer progression/regression. Notably, TEX have been implicated in inhibiting functions of immune cells either by inducing apoptosis of CD8^+^ anti-tumor effector cells (20, 21), down-regulating signaling in T and NK cells (22), inhibiting cytotoxicity (4, 23), or up-regulating functions of suppressor cells, Treg, and/or MDSC (24–26). The role of plasma-derived exosomes in AML is consistent with down-regulation of NK activity, largely but not entirely due to the presence of membrane-bound TGF-β1 on these nanovesicles, as reported by us previously (4). It appears that TGF-β1 is carried by exosomes isolated from almost all AML patients’ plasma specimens we examined, and its exosomal expression levels vary in patients tested at diagnosis vs. those tested during or after CT. It also appears that exosomal TGF-β1 expression levels discriminate better than total exosomal protein levels among patients, especially at diagnosis and during consolidation CT. Further, patients in long-term clinical remission, who had no residual disease, generally had low exosomal TGF-β1 levels. Nevertheless, these patients’ exosomal TGF-β1 showed distinct profiles, an indication that the observed distinct forms of TGF-β1 in AML exosomes obtained from the plasma of patients in CR might have prognostic significance. Although exosomal TGF-β1 is detected in western blots mainly in the latent form (LAP), it signals via the SMAD pathway when delivered to cells, as previously shown by us (4). In studies with human NK cells co-incubated with TGF-β1^+^ exosomes isolated from plasma of AML patients, we demonstrated that their biological activity was mediated mainly by the mature form of TGF-β1. This implies that exosomal latent TGF-β1 is being converted into a mature, active form that mediates biological activity when delivered to cells. The pro-peptide and LAP forms of TGF-β1 abundantly present in isolated AML exosomes, but not NC exosomes may be ready for utilization and their processing to active TGF-β1 may be a link to TGF-β1-mediated suppression of immune cells seen in AML (4). As the expression pattern of TGF-β1 on exosomes is related to their biological activity, it might serve as a biomarker of response to therapy and might reflect the presence/absence of residual disease after therapy. Future profiling of serially harvested exosomes when accompanied by functional analysis and correlations with the relevant clinical data should confirm the value of monitoring of the exosomal molecular content in AML. Already there is evidence (27) that profiling the exosomal mRNA content from treatment-naïve AML patients can reveal the presence of transcripts relevant to AML prognosis (FLT3-ITD, NPM1), treatment (FLT3-ITD, IGF-IR, CXCR4), and the leukemic niche function (IGF-IR, CXCR4, MMP4).

Perhaps the most critical question to be asked is whether AML plasma-derived exosomes originate from leukemic blasts or from another cellular source, such as transfusions of platelets or red blood cells these patients receive. This is of great importance, because if the former is true, then blast-derived exosomes should qualify as very promising biomarkers of AML progression and outcome. Our preliminary data based on immune capture of AML exosomes with anti-CD34 antibodies suggest that TGF-β1 is carried by CD34^+^ exosomes and also by CD34^−^ exosomes. Regardless of their cellular source, however, TGF-β^+^ exosomes exert profound biological effects on functions of immune and non-immune cells, as previously reported (27–31). As such, they are likely to influence cancer progression and might track with responses to therapy. Further studies are warranted to adequately assess the role of exosomes as potential diagnostic or prognostic biomarkers in AML.

## Author Contributions

Chang-Sook Hong and Laurent Muller performed the experiments. Michael Boyiadzis and Theresa L. Whiteside developed the concept, analyzed the data, and drafted the manuscript. All authors reviewed and approved the submitted manuscript.

## Conflict of Interest Statement

The authors declare that the research was conducted in the absence of any commercial or financial relationships that could be construed as a potential conflict of interest.
